# Case report: Clinical features and prognosis of two Infants with rhabdomyosarcoma of the tongue

**DOI:** 10.3389/fonc.2022.934882

**Published:** 2023-01-04

**Authors:** Peiyi Yang, Na Xu, Yan Su, Chao Duan, Shengcai Wang, Libing Fu, Tong Yu, Ruolan Guo, Xiaoli Ma

**Affiliations:** ^1^ Medical Oncology Department, Pediatric Oncology Center, Beijing Children’s Hospital, Capital Medical University, National Center for Children’ s Health, Beijing, China, Beijing Key Laboratory of Pediatric Hematology Oncology, Key Laboratory of Major Diseases in Children, Ministry of Education, Beijing, China; ^2^ Department of Otolaryngology Head and Neck Surgery, Beijing Children’s Hospital, Capital Medical University, National Center of Children’s Health, Beijing, China; ^3^ Department of Pathology, Beijing Children’s Hospital, Capital Medical University, National Center of Children’s Health, Beijing, China; ^4^ Department of Image Center, Beijing Children’s Hospital, Capital Medical University, National Center of Children’s Health, Beijing, China; ^5^ Medical Genetics Center of Beijing Pediatric Research Institute, Beijing Children’s Hospital, Capital Medical University, National Center of Children’s Health, Beijing, China

**Keywords:** infant, spindle cell rhabdomyosarcoma, tongue, susceptibility gene, case report

## Abstract

**Background:**

Rhabdomyosarcoma (RMS) is the most common soft tissue tumor in children, and its most common pathological types include embryonal RMS and alveolar RMS. In contrast, spindle cell RMS (SRMS) is a rare type. Moreover, the tongue is a rare primary site of RMS, and infancy is a rare age at onset.

**Case presentation:**

Two infants were diagnosed with lingual RMS at 3 and 5 months after birth, respectively, and were admitted to Beijing Children’s Hospital. The pathological type in both cases was SRMS. Both were classified as low-risk and were treated with surgery and chemotherapy. Case 1 was in complete remission at the latest follow-up, and Case 2 had a relapse 10 months after stopping chemotherapy, achieving complete remission after the multimodal treatment of chemotherapy, surgery, and radiotherapy. The venous blood gene test of the two infants did not indicate a pathogenic mutation or a possible pathogenic mutation related to RMS. In Case 1, variants of the *CDK4* and *BRCA1* genes, both with unknown significance and a possible relation to RMS, were detected. In Case 2, three gene variants of unknown significance that were possibly associated with RMS—*TRIP13*, *APC*, and *RAD54L*—were identified.

**Conclusion:**

Lingual RMS in infants is rare. Its clinical manifestations lack specificity, and early recognition is complex. The success and timing of local treatment are important prognostic factors. Genetic testing may be helpful for the early detection of tumor susceptibility and the estimation of prognosis.

## Introduction

Rhabdomyosarcoma (RMS) is the most common soft tissue tumor in children, accounting for approximately 6.5% of childhood tumors. Its most common primary sites are the head, neck, and genitourinary system, followed by the limbs and trunk, among others (1). RMS of the oral cavity accounts for 10%–12% of all head and neck RMSs (2). The tongue, palate, and cheek are the most common sites in the oral cavity, among which the tongue is relatively rare (3). The most common pathological types of RMS include embryonal RMS and alveolar RMS, whereas spindle cell RMS (SRMS) is rare and is currently incorporated with sclerosing RMS (ScRMS) as an independent subtype of RMS (4), accounting for approximately 5%–10% of all pathological types (5). Meanwhile, a previous study reported that infancy is a rare age of onset for RMS (6). The median age of 213 children with RMS reported by Beijing Children’s Hospital was 48 months, and infants accounted for 7% of the cases (7). Herein, we report two cases of SRMS originating from the tongue. Analyzing the clinical features of the cases and exploring the susceptibility genes possibly associated with the disease may improve our understanding of SRMS of the tongue in infants.

## Case description

### Case 1

A 4-month-old girl was admitted to our hospital with a mass of the tongue that had been previously resected. The mass was 3–4 mm in diameter and was discovered on the left lingual margin of the tongue more than 1 month after birth. Consultation with a doctor was not sought, and the tumor gradually increased to approximately 6–8 mm in diameter 2 months later. The tumor did not considerably affect drinking. Subsequently, she was admitted to a local hospital and was preliminarily diagnosed with “mucoceles on the left margin of the tongue”, which was surgically resected. The pathologic diagnosis was highly differentiated SRMS. The neoplastic cells were short and fusiform, with atypia and mitosis, and they grew infiltratingly into the surrounding muscle tissue. The immunohistochemical results were Desmin (+), Myogenin (+), MyoD1 (+), Ki-67 (10%), Myoglobin (+), CD34 (+), S-100 (−), SMA (−), and TFE3 (−).

No abnormalities in the birth, maternal pregnancy, or family histories of the patient were noted.

Physical examination revealed some tissue missing at the left lingual margin related to surgery, and no other abnormality was observed. Her body weight was 6.9 kg at admission (age 4 months old), which was normal. Laboratory examinations, including the blood routine, C-reactive protein, electrolyte, and lactic dehydrogenase levels, liver and kidney function, and coagulation function, revealed no abnormalities. Enhanced magnetic resonance imaging (MRI) indicated that the soft tissue in the right palatal and parapharyngeal spaces was slightly thicker than that in the contralateral space, and the strengthening was slightly more evident. Chest computed tomography (CT), abdominal ultrasound, cranial MRI, a bone marrow smear, and a bone scan revealed no metastatic lesions. The infant was finally diagnosed with SRMS of the lingual surface. The clinical TNM staging was T1aN0M0, and the Intergroup Rhabdomyosarcoma Studies (IRS) staging was II; thus, the tumor was classified as low risk.

The infant started receiving chemotherapy after she was diagnosed at our hospital, more than 1 month postoperatively. The treatment included four courses of vincristine, actinomycin D, and cyclophosphamide (the VAC regimen), followed by four courses of vincristine and actinomycin D (the VA regimen). Subsequently, chemotherapy was stopped, and she did not receive radiotherapy. At her last review, she had been followed up for 26 months, and the tumor was in complete remission without metastasis. She had normal height, weight, and language development, and she could eat normally without any abnormal taste.

### Case 2

**Figure 1:**
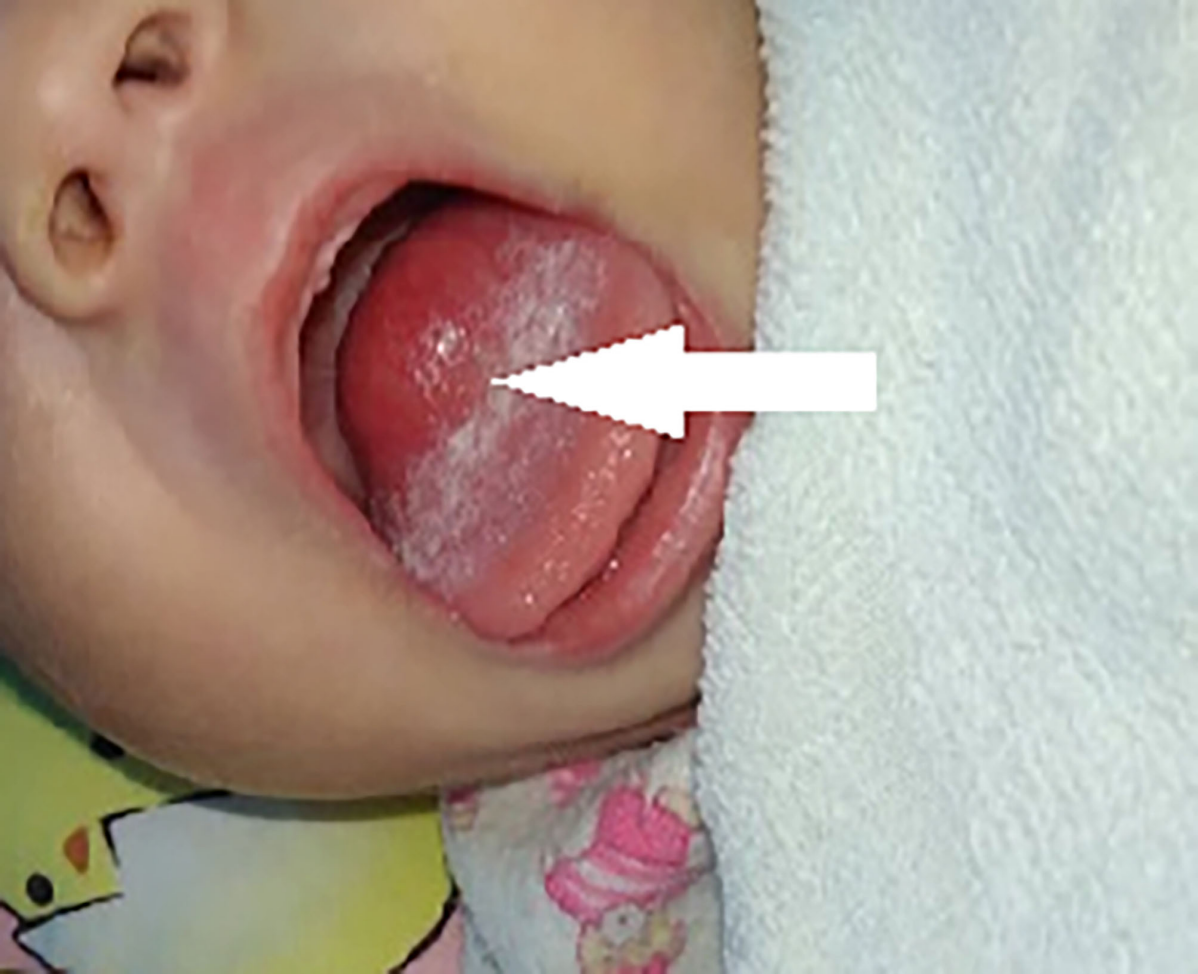
Case 2, tongue appearance at diagnosis.

A 3-month-old girl was admitted to our hospital after experiencing choking for 2 months. A mass at the root of the tongue was detected upon physical examination (**Figure 1**) in a local hospital, and the MRI revealed a mass measuring 23 mm × 17 mm × 18 mm. The mass led to difficulty drinking and slow weight gain. Her weight was 3.35 kg at birth and barely increased in the first month after birth. She was preliminary diagnosed with a “tumor of the root of the tongue of unknown nature”, which was also surgically resected. Histological examination revealed a mesenchymal spindle cell tumor, which was suspected to be a fetal rhabdomyoma. She was admitted to our hospital 2 months postoperatively. A pathological consultation in our hospital revealed an SRMS. The neoplastic cells are composed of spindle cells arranged in bundles or sheets, with mild atypia. Mitotic images were visible, and the neoplastic cells indicated infiltrative growth. The immunohistochemical results were as follows: desmin (+), myogenin (Scattered+), MyoD1 (+), SMA (+), and CD117(−). Fluorescence *in situ* hybridization (FISH) of *NCOA2* gene-related translocations was negative.

No abnormalities in the birth, maternal pregnancy, or family histories of the patient were noted.

Additionally, no evident abnormalities were detected upon physical examination. The body weight was 5.5 kg at admission (age 3 months old). Laboratory examinations, including the blood routine, C-reactive protein, electrolyte, and lactic dehydrogenase levels, liver and kidney function, and coagulation function, also revealed no abnormalities. The maxillofacial-enhanced MRI performed in our hospital indicated that the base of the tongue was thick, and uneven lamellar enhancement of a fat-saturated T2 signal was observed; this was considered a postoperative change. Chest CT, abdominal ultrasound, cranial MRI, bone marrow smear, and bone scan indicated no metastatic lesions. Finally, the infant was diagnosed with SRMS of the tongue, with a clinical TNM staging of T1aN0M0 and an IRS staging of I; the tumor was thereby classified as low risk.

**Figure 2:**
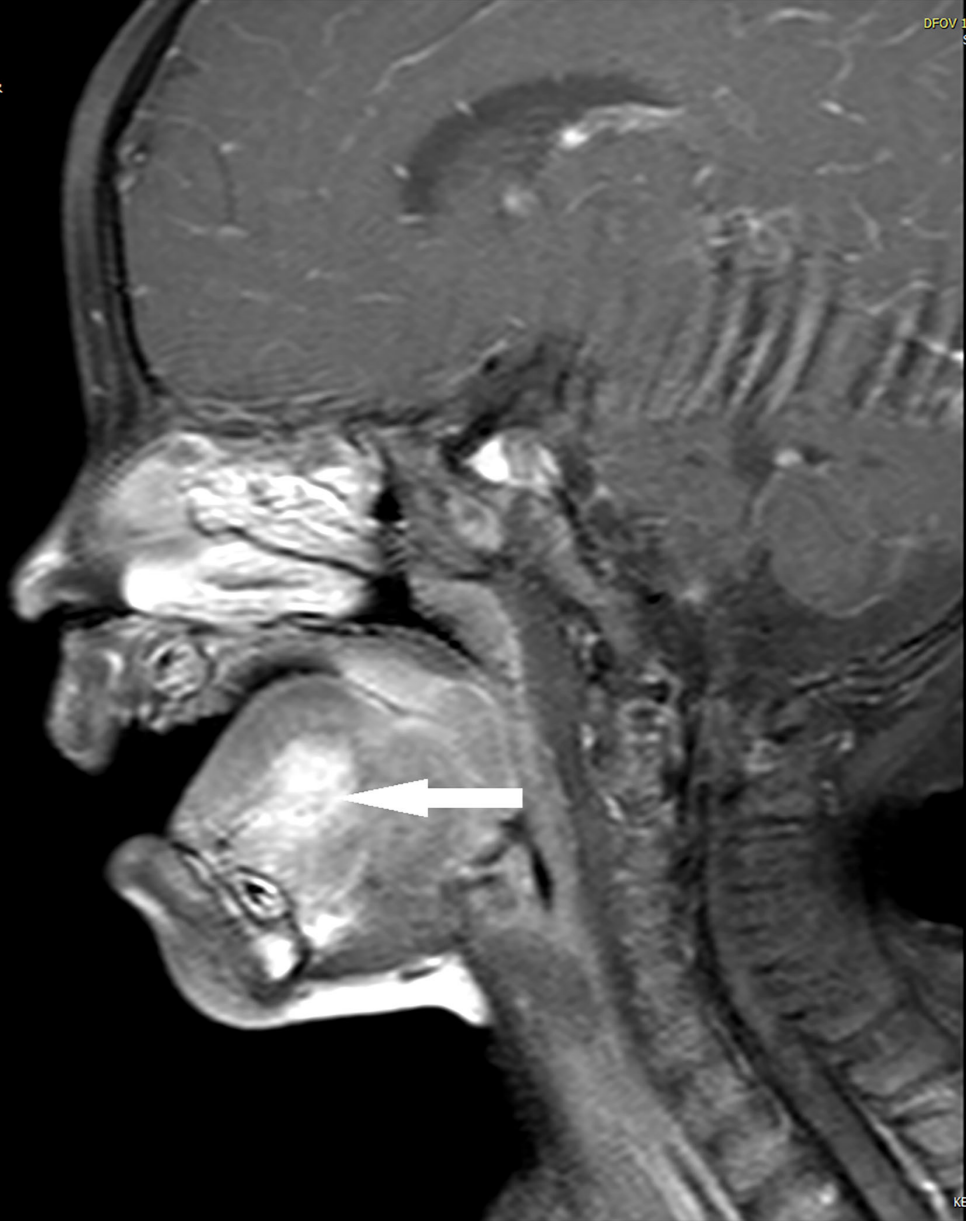
Case 2, tumor in magnetic resonance imaging at relapse (sagittal).

**Figure 3:**
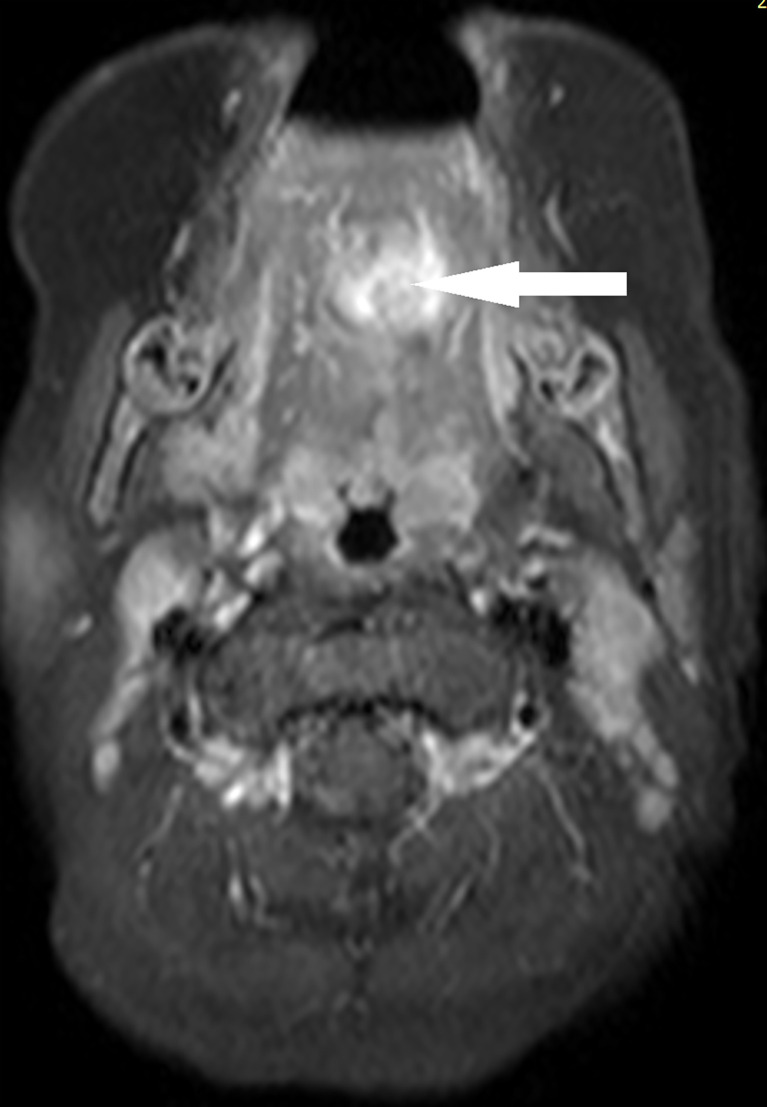
Case 2, tumor in magnetic resonance imaging at relapse (axial).

The patient started receiving chemotherapy once she was diagnosed, which began 2 months postoperatively. The chemotherapy included four courses of the VAC regimen, followed by four courses of the VA regimen. She did not receive radiotherapy. The maxillofacial-enhanced MRI performed 10 months after the completion of chemotherapy indicated that the range of nodular and patchy fat-saturated T2 signal in the tongue’s body and base increased, demonstrating uneven and obvious enhancement with an unclear boundary involving the geniohyoid, left geniohyoid, and mylohyoid muscles (**Figures 2**, **3**). Bilateral cervical lymph nodes were partially enlarged with enhancement. After a multidisciplinary consultation, tumor recurrence was considered. Consequently, chemotherapy was initiated again, which alternately comprised a vincristine, doxorubicin, and cyclophosphamide regimen and an IE (icyclophosphamide and etoposide) regimen, for a total of 11 courses. After the 11 courses ended, surgery was performed, and the tumor was totally resected. Postoperative pathology indicated no residual tumor cells. Subsequently, chemotherapy with vincristine and irinotecan for two courses was provided, followed by proton radiation therapy (total dose: 41.4 Gy) administered 23 times. After 31 months of follow-up, the tumor was in complete remission without metastasis. Fortunately, the patient also had normal height, weight, and language development, and she could eat normally without any abnormal taste at the last follow-up.

### 2.3 Single-gene disease exome–custom capture sequencing

With the informed consent of the patients’ parents, exome–custom capture sequencing was performed to identify single-gene diseases. Target sequences were captured, sequenced, and analyzed in the exon coding region of more than 6,100 genes known to be associated with human diseases, as well as in the 5-bp region of adjacent introns (excluding the untranslated region). Peripheral venous blood samples of patients and their parents were collected, and genomic deoxyribonucleic acid was extracted to classify variant pathogenicity. The classification of variations was as follows: pathogenic, possible pathogenic, unknown significance, possible benign, and benign variations.

The results revealed no pathogenetic or possible pathogenetic variation in the two cases, which was consistent with the genealogical inheritance pattern and related to the phenotype of the infants. In Case 1, the following two variants of unclear significance that are possibly related to the disease were detected: 1) heterozygous variation of *CDK4* (NM000075.3), named C. 806C > T (p.Ala269VAL), from the mother that is not included in the Clinvar/HGMD database and whose software forecasts are inconsistent; and 2) heterozygous mutation of *BRCA1* (NM007294.3), named C. 3350T > C (p.Val1117ala), from the father that is not included in the Clinvar/HGDD database and is predicted as benign by the software. In Case 2, the following three variants of unknown significance were detected and may possibly be associated with the disease: 1) heterozygous variation of *TRIP13* (NM004237.3), named C. 572C > T (p.Ala191Val), from the mother that is not included in the Clinvar/HGMD database and is predicted as deleterious by the software; 2) heterozygous variation of *APC* (NM000038.5), named C. 8437A > G (p.THR2813ALA), from the mother that is not included in the Clinvar/HGMD database and is predicted as inconsistent by the software; and 3) heterozygous variation of *RAD54L* (NM003579.3), named C. 689T > C (p.Val230Ala), from the father that is not included in the Clinvar/HGMD database and is predicted as deleterious by the software. In Case 2, the following three variants that are possibly associated with the disease were also detected: two *RAB3GAP2* variants and one *MMACHC* variant.

## 3 Discussion

The clinical manifestations of tongue RMS are its lack of specificity and complex recognition in its early stage, which may lead to misdiagnosis. In the early stage, the tumor expands and infiltrates the muscle from which it arises, presenting first as a well-demarcated nodule or polypoid lesion with a soft or gummy consistency. When these lesions grow rapidly, they may cause dyspnea, dysphagia, and cough, including acute respiratory obstruction (3). In Case 2, a mass in the tongue was identified because of the symptom of choking from drinking milk. The differential diagnosis of the mass of the tongue includes hemangiomas, fibromas, rhabdomyomas, lymphangiomas, and lingual thyroid (3, 8); primary lingual RMS is rare, and its definite diagnosis requires pathological examination. The two cases in this study were both finally diagnosed with RMS based on pathological results. In IRS I, II, and III, only seven cases of tongue RMS were registered, representing 0.34% of the IRS participants (9). Kebudi et al. summarized a total of 13 cases of lingual RMS reported in the English literature from 1973 to 2009, whose median age was 3.5 months, which was much younger than the median age of 5 years in children with RMS reported in the literature (8). Our two cases were initially considered a mucocele and a tongue root tumor of unknown nature at a local hospital, respectively. If the possibility of malignant tumors such as RMS is considered and further diagnosis and treatment are promptly performed after surgical resection, the optimal timing of treatment may be better identified.

Previous studies have demonstrated age as a strong, independent risk factor affecting the prognosis of RMS, even after considering other factors such as group, stage, and tumor histology. Infants aged <1 year and adolescents aged >10 years who were diagnosed with RMS had worse survival than children aged 1–9 years (10). Genetic differences in tumors, compliance with therapy, tolerance of therapy, differences in drug metabolism, or different levels of drug resistance within tumors may explain the effects of age on prognosis. Currently, the most recognized reason is that the complications and long-term adverse effects caused by surgery and radiotherapy are considered, and as a result, the local treatment intensity of infants is often low, leading to poor treatment outcomes (11). Among the 13 cases of lingual RMS reviewed by Kebudi et al., one 10-month-old girl received surgery after chemotherapy; the surgical margin was positive, and the RMS relapsed after 30 months of follow-up (8).

Previously, young children were assumed to have no tolerance for radiation therapy; however, when radiation is omitted, even in those with stage I disease, the risk of recurrence is high, with local recurrence being the most common, thus confirming the need for radiation therapy. Therefore, brachytherapy, proton therapy, intensity modulated radiation therapy, and other emerging precision radiotherapy methods may improve both the prognosis of the infant and radiotherapy tolerance (12). Case 2 was a patient with IRS stage I who did not receive radiotherapy and had a local recurrence 10 months later; she received proton therapy after the recurrence. At present, her condition is well controlled. Van Grotel et al. reported in 2003 that surgical debulking of the tumor combined with consecutive brachytherapy was used to treat a child who was resistant to chemotherapy, and the tumor was in complete remission after 3 years of follow-up with limited impact on eating and language function (13). Moreover, although the chemotherapeutic regimens in the two cases were consistent with those in the previous study, the timing of local and systemic treatment should be monitored. If the tumor is surgically removed first, chemotherapy should be started within 7 days postoperatively (1). Case 2 started chemotherapy 2 months after the first surgery; thus, the long interval may also be related to the recurrence.

SRMS is a rare type of RMS that mainly occurs in the paratesticular region and head and neck of children and adolescents. In the World Health Organization’s classification of soft tissue and bone tumors (2013 edition), SRMS and ScRMS were combined and classified as an independent subtype of RMS (4), accounting for approximately 5% to 10% of cases (5). Due to the similarities between SRMS and other spindle cell tumors, making an accurate diagnosis is very challenging. Therefore, using immunohistochemistry and even molecular genetic tests is vital to making a clear diagnosis and differential diagnosis of SRMS. MyoD1 was strongly positive in SRMS/SCRMS, desmin was diffused, and myogenin was weak (14); the pathological immunohistochemistry of our two cases was consistent with these characteristics. The current data support the notion that certain SRMS/ScRMS have a more aggressive clinical course with a reduction in long-term survival, and those in the head and neck region often exhibit extensive local recurrence (15). Owing to its genetic heterogeneity, its prognosis varies with different molecular and biological characteristics. Patients aged <1 year with *VGLL2*, *SBF*, *TEAD1*, and *NCOA2* gene fusions had a better prognosis, whereas patients with a *MYOD1* gene mutation demonstrated stronger tumor aggression and higher mortality, regardless of age. Considering that this distinct molecular subtype is characterized by more aggressive biologic behavior than other genetic subtypes of SRMS and ScRMS, the *MYOD1* genotype has been recommended to be used as a molecular marker in both subclassification and prognostication of SRMS/ScRMS (16). The two cases in this report involved SRMS in infancy, and the therapeutic effects were different. This may be related to the different molecular and biological characteristics. In Case 2, no *NCOA2* gene-related translocation was detected using FISH. However, the patient had no *MYOD1* gene mutation, making the consistency between this gene variation and prognosis difficult to judge. For patients with SRMS/ScRMS, these gene tests should be performed as much as possible to improve the accuracy of prognosis judgment and achieve stratified treatment.

Moreover, infantile-onset RMS may be associated with a potential cancer predisposition syndrome (CPS) (17). Local and foreign studies have reported that the occurrence and development of CPS-related RMS are related to different genetic characteristics (such as chromosomal translocation and heterozygosity deletion) and molecular pathway changes (such as some tumor suppressor genes and oncogenes) (18), which may cause chemotherapy intolerance or non-response, and some patients require long-term follow-up before diagnosis. The family syndromes associated with RMS include Li–Fraumeni syndrome (LFS), neurofibromatosis type 1, Rubinstein–Taybi syndrome, Beckwith–Wiedemann syndrome, and Costello syndrome (19). The pedigree of the patient with lingual RMS was reported by Kebudi et al. conformed to the characteristics of LFS (the proband was a 2-year-old boy with RMS, his aunt suffered from premenopausal breast cancer, and his uncle, aged <60 years, was diagnosed with prostate carcinoma) (8). RMS is the most common pediatric malignancy in a typical family with LFS (7), and the occurrence of LFS is related to the germ-line mutation of the tumor suppressor gene *TP53* (19). Additionally, in the case of lingual RMS reported by Van Grotel et al., after 15 years of follow-up, multiple skin lesions were observed on the patient’s right shoulder and back, and the biopsy pathology was basal cell carcinoma. Owing to the concomitant presence of a mandibular bone cyst, genetic testing was performed, and a *PTCH1* gene mutation was confirmed, leading to the diagnosis of Gorlin syndrome (20). Some genetic susceptibility syndromes can only be detected after long-term follow-up in some cases, according to the accompanying phenotype, so conducting long-term monitoring is necessary.

Both cases were diagnosed with RMS in infancy, so they underwent exome–custom capture sequencing of single-gene disease of the peripheral blood to determine any gene variations currently known to be associated with RMS. Among the mutations identified in the test that may be associated with RMS, the *CDK4* gene (Case 1) was located at 12q14, encoding the cell cycle regulator *CDK4*, and its amplification was observed in ARMS, breast cancer, non-small cell lung cancer, and glioblastoma multiforme (21). The *BRCA1* gene (Case 1), located at 17q21, is a tumor suppressor gene, and its mutation can be observed in breast cancer and ovarian cancer (22). The *TRIP13* gene (Case 2) is located on 5p15 and can be highly expressed in various malignant tumors, although no RMS has been reported (23). The *APC* gene (Case 2), located at 5q21-22, is also a tumor-suppressor gene, and its inactivation or mutation is associated with colorectal cancer, myeloma, RMS, and adenocarcinoma (24). The *RAD54L* gene (Case 2) was located at 1p32, and its mutation may be associated with breast cancer, colon cancer, lymphoma, and meningioma (25). The mutations in these genes in the two infants were classified as unknown mutations that were not included in the Clinvar/HGMD database. Although the occurrence of RMS in the two cases cannot be concluded as related to congenital genetic factors, considering their age at onset, primary site, and pathological type, continuing long-term follow-up is necessary in order to observe the outcome of the disease while summarizing other cases to reach a conclusion.

## 4 Conclusion

Lingual RMS in infants is rare, its clinical manifestations lack specificity, and its early recognition is complex. The success and timing of local treatment are important prognostic factors. Genetic testing may be beneficial to detect tumor susceptibility and prognosis early and to provide a theoretical basis for long-term follow-up of patients and their families.

## Data availability statement

The original contributions presented in the study are included in the article/supplementary material. Further inquiries can be directed to the corresponding author.

## Ethics statement

The studies involving human participants were reviewed and approved by the Ethics Committee of Beijing Children’s Hospital, Capital Medical University (2018-k-106). Written informed consent to participate in this study was provided by the participants’ legal guardian/next of kin. Written informed consent was obtained from the minor(s)’ legal guardian/next of kin for the publication of any potentially identifiable images or data included in this article.

## Author contributions

XM conceived the idea for this study and managed the cases. NX collected clinical data. PY and YS analyzed and interpreted the patients’ data. LF performed the histological examination. TY analyzed the MRI images. RG performed the single gene disease exome-custom capture sequencing. PY, NX, and XM were major contributors in the writing of the manuscript. All authors contributed to the article and approved the submitted version.
